# Effect of Discrimination on Presenteeism among Aging Workers in the United States: Moderated Mediation Effect of Positive and Negative Affect

**DOI:** 10.3390/ijerph17041425

**Published:** 2020-02-22

**Authors:** Jianwei Deng, Yuangeng Guo, Hubin Shi, Yongchuang Gao, Xuan Jin, Yexin Liu, Tianan Yang

**Affiliations:** 1 School of Management and Economics, Beijing Institute of Technology, Beijing 100081, China; 111605@bit.edu.cn (J.D.); guoyuangeng@foxmail.com (Y.G.); 18811363379@163.com (H.S.); gyc5896@163.com (Y.G.); jinxuanonly@163.com (X.J.); a1057499672@163.com (Y.L.); 2 Sustainable Development Research Institute for Economy and Society of Beijing, Beijing 100081, China; 3 Chair of Sport and Health Management, School of Management, Technical University of Munich, Uptown Munich Campus D, 80992 Munich, Germany

**Keywords:** discrimination, positive affect, negative affect, conscientiousness, presenteeism, public data

## Abstract

This study aimed to examine how perceived everyday discrimination influences presenteeism and how conscientiousness moderates the relationship between discrimination and positive affect among older workers. Structural equation modeling (SEM) was used to examine the mediating effect. The moderated mediation model was examined by PROCESS. The results of the final SEM model showed that discrimination was directly positively associated with presenteeism. Furthermore, positive affect was significantly inversely correlated with discrimination and presenteeism. In addition, negative affect was significantly positively correlated with discrimination and presenteeism. The significant indirect effect between perceived everyday discrimination and positive affect was significantly mediated by positive and negative affect. In addition, the results of the moderated mediation model indicate that positive affect was more likely to be influenced by perceived everyday discrimination among older workers with less conscientiousness, as compared with those with greater conscientiousness. To enhance work outcomes of aging workers in the United States, managers should foster highly conscientious aging workers, award those who are hardworking and goal-oriented, and combine personal goals and organizational goals through bonuses, holidays, and benefits. Policymakers should be mindful of the negative impact of discrimination on presenteeism and should target lowly conscientious older workers.

## 1. Introduction

Everyday discrimination is a critical concern because of the great burdens it exerts on societies [1,2] and individuals [3,4,5,6,7] worldwide. Everyday discrimination can be defined as chronic and routine unfair treatment that an individual encounters in everyday life—discrimination based on their actual or perceived characteristics, such as race, gender, sexual orientation, age, immigration status, income, medical condition, and mental or physical disability. It can be conveyed through opinions, attitudes, and behaviors and can be measured by documenting objective events or relying on subjective perceptions of events [8,9]. Some researchers have argued that previous studies failed to adequately sample the range of discriminatory events [10], which could have led to underestimation of the prevalence and personal consequences of discrimination [10]. On the one hand, discrimination incidents are often ambiguous—that is, people cannot be sure whether mistreatment they receive is the result of their age or gender or some other reason [10]. In addition, some regular incidents may still have adverse effects even if people do not expressly report that they have been ‘discriminated against’ [10,11]. Everyday discrimination can be based on a number of factors [8], including gender [9,12], age [13,14], weight [15], and sexual orientation [16].

Because of the remarkable growth in the number of aging workers, research on everyday discrimination against aging workers is extremely important [17]. Every country in the world is experiencing growth in the number and proportion of older persons [18]. Globally, the population of adults aged 65 years or older is growing faster than all other age groups [18], which means that the aging population is becoming the main component of the labor force. According to data from World Population Prospects, by 2050, one in six people in the world will be older than 65 (16%), up from one in 11 in 2019 (9%). By 2050, one in four persons living in Europe and Northern America could be 65 or older [18]. Everyday discrimination has a serious impact on aging workers, as it could cause them to develop self-critical thoughts, including negative self-perceptions of aging, which, in turn, can lead to depression [19]. Discrimination against aging workers has resulted in systematic discrimination and is not conducive to the equal social status of older workers [20]. Because of the slower growth of the labor force, most aging countries aim to improve work enthusiasm and productivity among aging workers [21,22]. Therefore, to address these practical and theoretical research gaps, this study examined the impact of everyday discrimination on presenteeism mediated by positive and negative affect. Because multiple facets of personality may affect perceptions of discrimination responses [23] among people with varying personality traits, we also consider the moderating effects of conscientiousness. 

The present findings are noteworthy for several reasons. First, we focus on issues related to everyday discrimination of aging workers. The aging of labor forces is one of the most significant problems faced by organizations and governments and is attributable to decreases in human death and birth rates, increases in statutory retirement age, and a reduction in retirement savings [24]. The proportion of workers older than 55 years is rapidly increasing around the world [15]. With the threat of labor shortages and insufficient pension funds, retaining old employees—and maintaining their work enthusiasm and performance—is now an important goal in many developed countries [21,22]. Therefore, the selection of aging workers as a research focus is the first valuable contribution of this paper. Because potential loss of productivity is also an important effect of discrimination, collection of data on the impact of discrimination on presenteeism is the second contribution of this paper. In addition, affective event theory (AET) is usually used to assess how workplace events influence employees’ affective states and, thus, their behaviors. We originally used AET to evaluate how life events affect aging workers’ affects and, ultimately, result in presenteeism. Perception of everyday discrimination may lead to negative affects among aging workers, thus contributing to presenteeism. Alternatively, when moderated by conscientiousness, it might trigger positive affects among aging workers and alleviate worsening of work ability. Research on this topic is useful for alleviating implicit absence in the workplace and improving enterprise performance. These are the goals of this study.

## 2. Theoretical Background and Literature Review

### 2.1. Theoretical Background

AET [25] emphasizes the role of discrete events as proximal causes of affective reactions that influence the attitudes and behaviors of people. For instance, an affective reaction emerges from a dual-level cognitive appraisal process that starts with an initial evaluation of an event’s relevance to the individual [26]. The event shapes the intensity of the person’s subjective emotional reaction, and the initial appraisal contributes to further specific appraisals of the potential causes of an emotional experience, as well as those consequences attributed to the event. In addition, AET proposes that affective events can determine affective states [25]. If, for example, an employee is treated unfairly by a leader (regular event), it generates anger (affective state) and contributes to the employee’s work outcome (behavior). 

Because discrete emotions endorsed at workplaces are essential to productivity, AET has been used as an effective analytic framework that can explain how employee output can be improved by managing their emotional labor [26]. Inspired by AET, Lam (2012) considered the integrative context of workplace environments, events, affect, attitudes, and behaviors, which includes the work environment (supervisory support), regular work events (interactional justice), and affective (unhappiness, anger, tiredness, anxiety, and hopelessness), and attitudinal (job satisfaction) and behavioral (emotional labor, service quality, and turnover) reactions [26]. As compared with affective events in the workplace, perceived everyday discrimination can be regarded as a kind of affective event in daily life, since it can lead to emotions such as depression [27,28,29].

**Figure 1:**
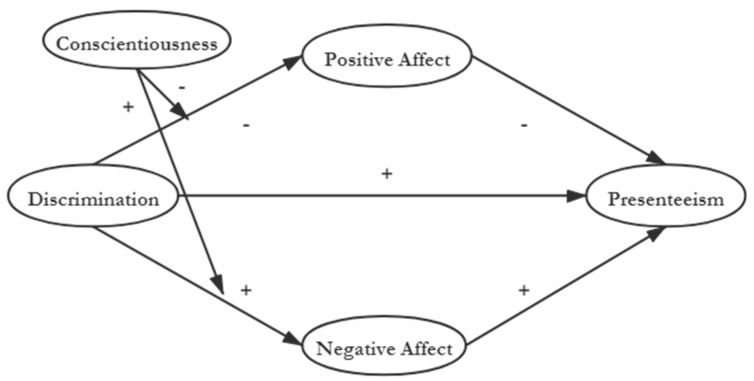
Proposed model of the relationship between discrimination, presenteeism, and positive and negative affect shows the effect of mediator resources, with conscientiousness as moderator.

Using AET and the theoretical framework of Lam [26], we propose a model (Figure 1) that is broadly consistent with AET [25]. This model posits that everyday discrimination (regular event) can influence positive and negative affects (affective states), which might diminish enthusiasm for work and contribute to presenteeism (affect-driven behavior).

### 2.2. Perceived Everyday Discrimination and Positive and Negative Affect

Everyday discrimination can be defined as the chronic and regular unjust treatment an individual experiences in daily life—discrimination based on their actual or perceived characteristics, such as gender [9], sexual orientation [16], age [13], and weight [15]. Conveyed by words, attitudes, and behaviors, everyday discrimination is normally measured by means of documentation or by assessing subjective perceptions of objective events [8,9]. 

As the construct of affect developed, affects captured by individuals were usually classified as positive affect and negative affect [30,31], because they were regarded as two relatively independent processes. Specifically, negative affect was defined as the extent to which a person experiences subjective distress, unpleasant engagement, and emotional pain [31], while positive affect—typically featuring happiness, excitement, or contentment—was a mental state best characterized by enjoyable engagement with the environment [32,33]. The AET theory recognized that affect itself was multidimensional, which meant people could feel angry or frustrated (negative affect) as well as proud or joyful (positive affect) at the same time [25].

AET further claimed that negative affective states were only present when a negative event (e.g., perceived everyday discrimination) occurred [25], but changes in positive affect attributable to regular events (e.g., perceived everyday discrimination) were not asymmetric with those of negative affect [25]. For instance, higher perceived discrimination derived from characteristics like race [34], gender [35], and weight [15] was found to be related to higher negative affect and lower positive affect. Based on previous studies, this paper attempts to study the different impacts of perceived everyday discrimination on positive and negative affect. Thus, we suggest that perceived everyday discrimination may lead to an upshift in negative affect (e.g., angry, guilty) and a downshift in positive affect (e.g., joyful, content), as indicated by two hypotheses:

**H1:**  *Perceived everyday discrimination is positively associated with negative affect*.

**H2:**  *Perceived everyday discrimination is inversely associated with positive affect*.

### 2.3. Perceived Everyday Discrimination and Presenteeism

As a representative counterproductive behavior and a crucial outcome variable of perceived everyday discrimination, presenteeism has received little attention until recently [36]. Definitions of presenteeism vary between studies [37] and are evolving, and there has been no unified definition of presenteeism, as the three main lines of thought are developing independently and are used in parallel [38]. The first, European, line [37] defines it as the act of attending work while ill and focuses on its antecedents and consequences [39,40,41]. The second line, developed by North American researchers, [37] defines it as a measurable loss of productivity attributable to attending work with health problems [42,43]. The third line defines presenteeism as being physically present but functionally absent [44], which is not strictly restricted to illness.

The abovementioned three lines have different focuses. From our perspective, potential productivity loss results from various causes, including health and reasons unrelated to illness [45,46,47,48]. In the present study, we define presenteeism, broadly, as potential productivity loss due to health or other events that distract employees from their full work capacity [45,46,47,48].

Although many determinants of presenteeism have been fully examined, only a few studies have investigated perceived everyday discrimination. As a kind of relational demand [36] and a psychological factor leading to potential productivity loss in the workplace [49], perceived everyday discrimination directly and indirectly leads to presenteeism by influencing the decision to report to work or not when ill [36] and by increasing the risks of physical and mental health problems (i.e., backache, muscle pain, stomachache, overall fatigue, headache, anxiety/depression, sleeping problems, and injury) [50,51,52,53]. AET considers perceived everyday discrimination as a regular event that can influence the affective states of employees and thus, their enthusiasm for work. This affect-driven behavior ultimately links it to presenteeism. Thus, we hypothesized that discrimination would affect presenteeism among aging workers.

**H3:**  *Perceived everyday discrimination is directly positively related to presenteeism*.

### 2.4. Positive Affect, Negative Affect, and Presenteeism

Researchers now recognize the important role of positive and negative affect in the workplace [54,55]. With respect to AET, affective states of labor might influence workers’ presenteeism and other work-related problems [56]. Positive affect and negative affect potentially influence physical pain, which is then linked to productivity loss [57]. For example, sleep disturbance indirectly predicts pain interference via negative and positive affect [58]. Positive affect can serve as a buffer against adverse pain outcome [59], while negative affect may influence pain by interfering with sleep [58]. Moreover, positive affect and negative affect were frequently used as mediators in research models, because of their close inter-relationship [60,61,62,63].

As to AET, different affective reactions can have different behavioral implications [25]; thus, positive and negative affect may have different effects on presenteeism. Positive affect was reported to be negatively related to presenteeism, whereas negative affect was positively related to presenteeism [64]. Employees with negative affect may have difficulty enacting constructive coping behaviors [65] and may instead opt for emotion-focused coping, which involves impulsive and destructive responses [66] that could potentially culminate in presenteeism [67,68]. In contrast, positive affect facilitates creative problem-solving and flexible cognitive processing [69] and often occurs with a promotion regulatory focus [70], which itself may be negatively related to presenteeism. In sum, theoretical and empirical evidence supports the linkage of affect and performance. Everyday discrimination (regular event) can result in downshifting of positive affect and upshifting of negative affect (affective states), which might thereby diminish enthusiasm for work, thus contributing to presenteeism (affect-driven behavior).

**H4:**  *Positive affect is inversely related to presenteeism*.

**H5:**  *Negative affect is positively related to presenteeism*.

**H6:**  *Positive affect mediated the relation of perceived everyday discrimination with presenteeism, and negative affect mediated the relation of perceived everyday discrimination with presenteeism*.

### 2.5. Conscientiousness as a Moderator

Research on the “Big 5” personality traits suggests that personality is strongly related to mood [71,72,73,74]. Several studies analyzed the “Big 5” personality traits, both together [75,76,77] and singly or in pairs, as moderators in research models [78,79]. In the “Big 5” model of personality, conscientiousness is conceptualized as a higher-order personality trait that subsumes lower-order traits, including competence and achievement, orderliness, and self-control or deliberation [80,81]. Conscientiousness is arguably the most significant personality trait [81] and is commonly regarded as a personal resource that helps individuals mitigate the impacts of stress [79]. Recent meta-analyses found that conscientiousness is inversely associated with negative affect [82]. Individuals with high conscientiousness work harder and are more likely to obey rules [83]; when they perceived discrimination, their capacity for emotional recovery is stronger [24]. Studies have explored the moderating effect of conscientiousness on the relation between negative events and negative affect. Chi and Ho (2014) [74] confirmed that conscientiousness among workers moderates negative affect attributable to leaders’ complaints. Thus, we assumed that perceived everyday discrimination greatly influences affects among aging workers with low conscientiousness.

**H7:**  *Conscientiousness negatively moderates the mediating effects of perceived everyday discrimination on positive affect and positively moderates the mediating effects of perceived everyday discrimination on negative affect*.

## 3. Materials and Methods

### 3.1. Sample and Data Source

Cross-sectional data for the current study were collected from respondents participating in the 2016 wave of the Health and Retirement Study (HRS). The HRS was sponsored by the National Institute on Aging (grant number NIA U01AG009740) and was conducted by the University of Michigan in the United States. 

The HRS began in 1992 and now includes more than 26,000 respondents. People older than 50 years were recruited by means of multistage sampling for participation in biennial surveys that assess the characteristics of the aging population [84,85]. To keep the sample representative, HRS researchers add new respondents every 6 years. In 2006, the HRS added a Participant Lifestyle Questionnaire (PLQ) to their core biennial survey; the PLQ was administered to a random 50% of core panel participants. Additional details regarding the design of the HRS are available elsewhere (http://hrsonline.isr.umich.edu).

In the 2016 wave of HRS, data from the Lifestyle and Behavior Questionnaire (LBQ) were available for 8388 participants. Among these participants, 2152 (25.7%) were currently employed and answered at least one question on the “LBQ 2016”. In this study, we defined aging workers as those older than 55 years. Among employed participants, 2006 (93.2%) were aged 50 to 59 years. The percentage of those with missing data was less than 10%. Because presenteeism is only observed among currently employed participants, only data from the 2152 currently working participants were analyzed in this study.

### 3.2. Measures

*Perceived Everyday Discrimination.* Discrimination was assessed by using a scale of global perceptions of everyday unfair treatment [86]. This scale consists of nine items and measures encounters with discrimination in the respondent’s day-to-day life. Representative items include, “How often have you been treated with less courtesy than others?” Items are asked of respondents regardless of the attribution for unfair treatment. Responses are scored on a 5-point Likert scale ranging from 1 = almost every day to 6 = never. In these analyses, the mean of the total scale score was used; higher scores indicate lower discrimination. Data for this scale in 2016 were found to have high reliability (Cronbach α = 0.83)

*Presenteeism.* Presenteeism was assessed by using a reliable and validated instrument—the Perceived Ability to Work Scale (PAWS)—to analyze a job’s physical, mental, and interpersonal demands [87,88]. We believe this instrument is a valid measure of presenteeism because it estimates perceived productivity losses [88] and has been frequently used in previous studies [45,46,47,48]. Because several recent studies have defined presenteeism as potential productivity loss in the workplace attributable to health or other events [41,42,43,44], we used this definition in the present study. This scale consists of four items, and only currently working participants were asked to respond. Representative items include “Thinking about the interpersonal demands of your job, how do you rate your current ability to meet those demands?” Responses are scored on an 11-point Likert scale ranging from 0 = “cannot currently work at all” to 10 = “work ability is currently at its lifetime best”. In these analyses, the mean of the total scale score was used; higher scores indicate greater current ability to work. Presenteeism was measured by using the PAWS, which is a reliable tool for measuring perceived productivity loss and was reported to have acceptable psychometric characteristics in previous empirical studies and in the US Health and Retirement Surveys [45,89]. The data for the scale in 2016 were reported to have high reliability (Cronbach α = 0.89). Higher scores indicate lower presenteeism and higher perceived ability to work.

*Positive and Negative Affect.* These scales assess positive and negative dimensions of emotional (hedonic) well-being. In 2008, researchers started to choose most of the 25 items to assess positive and negative affect from the Positive and Negative Affect Schedule—Expanded Form (PAN AS-X) [31]. Some items were obtained from the work of other researchers in this field [90,91]. The scale consists of 25 items. Positive affect was defined as determined, enthusiastic, active, proud, interested, happy, attentive, content, inspired, hopeful, alert, calm, and excited. Data for the positive affect scale in 2016 were reported to have high reliability (Cronbach α = 0.93). Negative affect was defined as afraid, upset, guilty, scared, frustrated, bored, hostile, jittery, ashamed, nervous, sad, and distressed. Data for the negative affect scale in 2016 were reported to have high reliability (Cronbach α = 0.90). Responses were scored on a 5-point Likert scale ranging from 1 = very much to 5 = not at all. In these analyses, the mean of the total scale score was used; higher scores indicate lower positive as well as lower negative affect. 

*Conscientiousness.* The conscientiousness trait was evaluated on a scale of 1 = a lot to 4 = not at all. Higher values indicate greater conscientiousness. Our research uses the “Big 5” Personality Traits scale [92]. Conscientiousness was based on the following traits: organized, responsible, hardworking, careful, and thorough. Data for this scale in 2016 had satisfactory reliability (Cronbach α = 0.67). Higher scores indicate lower conscientiousness.

*Demographic Factors.* Age was defined as age in years at baseline. Gender was coded as 1 = men and 2 = women. 

### 3.3. Statistical Analysis

SPSS 24.0 (IBM Corp.; Armonk, NY, USA) and AMOS 24.0 (IBM Corp.; Armonk, NY, USA) was used for statistical analysis comprising descriptive analysis and path analysis. Missing values for observed indicators were imputed by using expectation maximization (EM) implementation of maximum likelihood. Structural equation modeling (SEM) was used to examine associations among perceived everyday discrimination, positive affect and negative affect, and presenteeism. The moderated mediation model was used to examine the moderated mediation effects of conscientiousness. AMOS 24.0 and PROCESS Model 7 in SPSS were used for the mediation analysis and moderated mediation analysis, respectively.

Before SEM, correlation analysis was used to determine the significance of correlations between perceived everyday discrimination, positive affect and negative affect, conscientiousness, and presenteeism. SEM analysis was used to distinguish relationships between discrimination, positive and negative affect, and presenteeism. The criteria used to evaluate good global fit were a root mean square error of approximation (RMSEA) less than 0.08 and goodness of fit index (GFI), normed fit index (NFI), comparative fit index (CFI) and Tucker–Lewis index (TLI) values of 0.90 or higher.

A non-parametric resampling procedure was used to test mediation with SPSS INDIRECT Macros Model 4, as recommended by Preacher and Hayes (2004) [93]. This was considered the most straightforward bootstrapping method to acquire confidence intervals for indirect effects [94]. Mediation was proved if the indirect effect was significant and the confidence interval did not include zero [95]. 

The moderated mediation model was tested with PROCESS Model 7 in SPSS. When significant mediation was established, conditional indirect effect procedures were used to determine whether mediation depended on the level of the theoretically proposed moderator (i.e., conscientiousness) [96,97]. One thousand bootstrapping re-samples generated 95% confidence intervals, and the moderated mediation model was tested to determine if the conditional mediation model was significant for presenteeism [97].

## 4. Results

### 4.1. Preliminary Analysis

Among the participants, 937 (43.6%) were male and 1215 (56.4%) were female; 146 (6.8%) were younger than 50 years, 533 (24.8%) were aged 51 to 55 years, 599 (27.8%) were aged 56 to 60 years, 457 (21.2%) were aged 61 to 65 years, 212 (9.9%) were aged 66 to 70 years, and 204 (9.5%) were older than 70 years. Detailed results are shown in Table 1.

### 4.2. Means (SD) for Discrimination, Conscientiousness, Positive and Negative Affect, and Presenteeism

Table 2 shows the mean (SD) values for discrimination, conscientiousness, positive and negative affect, and presenteeism. The means for the five discrimination items were very high, but the range was considerable. The means ranged from 4.81 (“You receive poorer service or treatment than other people from doctors or hospitals”; SD = 1.28) to 5.75 (“You are treated with less courtesy or respect than other people”; SD = 0.62). The mean for the positive affect items was lower than that for the negative affect items. The fifth positive affect item (“happy”) had the lowest score (M = 1.99, SD = 0.94), and the twentieth item (“excited”) had the highest score (M = 2.61, SD = 1.09). The ninth negative affect item (“ashamed”) had the highest score (M = 4.68, SD = 0.71), and the fifth item (“frustrated”) had the lowest score (M = 3.56, SD = 1.11). The means for conscientiousness ranged from 2.77 (“impulsive”, SD = 0.86) to 3.78 (“hardworking”, SD = 0.49; “responsible”, SD = 0.75). The means for the four presenteeism items were extremely high and ranged from 8.49 (“Thinking about the physical demands of your job, how do you rate your current ability to meet those demands?” SD = 1.80) to 8.70 (“Thinking about the mental demands of your job, how do you rate your current ability to meet those demands?” SD = 1.57).

### 4.3. Model Fit

As shown in Table 3, despite a marginal violation (the average proportions of indicator variance extracted (AVE) for negative affect and conscientiousness did not yield an acceptable fit), the scores for composite reliability (CR) suggest acceptable reliability.

### 4.4. Pearson Correlations between Discrimination, Conscientiousness, Positive and Negative Affect, and Presenteeism.

Correlation coefficients (r) show correlations between items within the same construct (Table 4). Positive affect was significantly negatively correlated with negative affect (r = −0.45). Positive affect and negative affect yielded different outcomes: positive affect was significantly negatively correlated with discrimination and presenteeism (r = −0.22 to r = −0.34), while negative affect was significantly positively correlated with discrimination and presenteeism (r = 0.41 to r = 0.28). There was a significant positive correlation between discrimination and presenteeism (r = 0.22).

### 4.5. SEM Model

**Figure 2:**
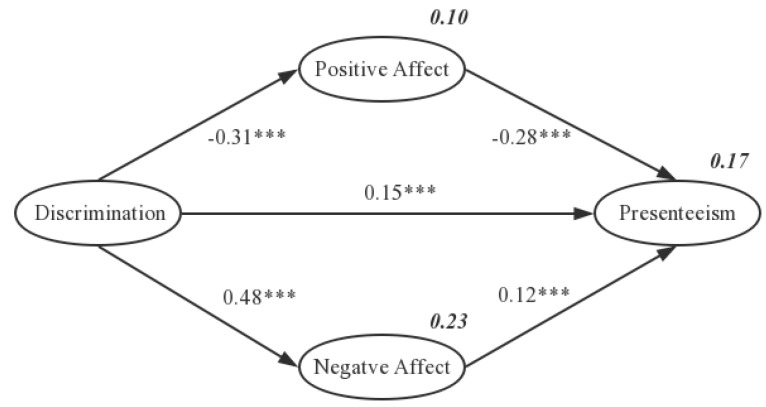
Mediator model of how discrimination influences presenteeism via positive and negative affect. (The numbers not in bold are standardized regression coefficients; numbers in bold explain variability; chi-square = 3293.822; degrees of freedom = 480, *p* < 0.001; root mean square error of approximation = 0.052; normed fit index = 0.908; comparative fit index = 0.920; goodness-of-fit index = 0.913; *** *p* < 0.001).

We used SEM to examine the mediating role of positive affect and negative affect on the influences of perceived everyday discrimination and presenteeism. Before SEM, analysis of the measurement model showed that our model fit the data well: the values for the goodness-of-fit index and comparative fit index for each measurement model were all between 0.908 and 0.920. As shown in Figure 2, perceived everyday discrimination had a significant positive correlation with presenteeism (*β* = 0.15, SE = 0.030; *p* < 0.001), a significant negative correlation with positive affect (*β* = −0.31, SE = 0.084; *p* < 0.001), and a significant positive correlation with negative affect (*β* = 0.48, SE = 0.060; *p* < 0.001). Positive affect had a significant negative correlation with presenteeism (*β* = −0.28, SE = 0.048, *p* < 0.001), and negative affect had a significant positive correlation with presenteeism (*β* = 0.12, SE = 0.085; *p* < 0.001). Therefore, positive affect and negative affect mediate the influence of perceived everyday discrimination on presenteeism.

In SPSS, mediation analysis with model 4 of the PROCESS macro was used to determine whether positive affect and negative affect mediated the association between perceived everyday discrimination and presenteeism. This reasonable bootstrapping technique yielded confidence intervals for indirect effects. A significant indirect effect and mediation were considered present when the confidence interval did not include zero. The analysis showed that discrimination was related to lower positive affect, which in turn was related to greater presenteeism (point estimate of indirect effects = 0.1192, SE = 0.017, 95% BCa CI = 0.0877 to 0.1549). Similarly, discrimination was related to greater negative affect, which in turn was related to greater potential loss of productivity (point estimate of indirect effect = 0.0955, SE = 0.232, 95% BCa CI = 0.0496 to 0.1417).

### 4.6. Examination of Moderated Mediation Model

Because of theoretical considerations and the significant mediation model, we used SPSS PROCESS Macros Model 7 to examine the moderating role of conscientiousness in the process by which discrimination influences positive and negative affect. Moderated mediation was tested with PROCESS Model 7, with positive affect as the outcome variable. As shown in Table 5 the product term conscientiousness had a significant predictive effect on positive affect (B = 0.1023, *p* < 0.05). Hayes (2014) [96] and Hayes (2013) [97] maintain that a conditional indirect effect is present if the relationships between the independent variable and moderators are significant and the bootstrapping confidence intervals do not include zero.

**Figure 3:**
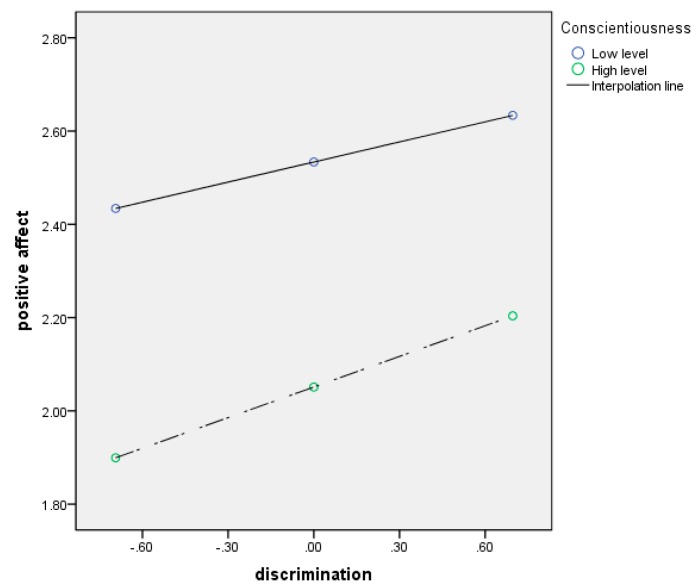
Moderating effects of conscientiousness in predicting positive affect.

As shown above, higher conscientiousness scores indicate lower conscientiousness; thus, the lower score for conscientiousness, below, indicates greater conscientiousness. The data shown in Table 5 suggest that conscientiousness significantly moderated the indirect effect of discrimination on positive affect. Specifically, the interaction effect between discrimination and conscientiousness increased as conscientiousness decreased. As shown in Figure 3 and Table 6, older workers with low conscientiousness were more likely to be affected by perceived everyday discrimination than were those with high conscientiousness. The effect on positive affect was strongest when conscientiousness was lowest.

## 5. Discussion

We used data from 2152 workers and affective event theory to examine how perceived everyday discrimination influences presenteeism and potential productivity loss through positive and negative affect and how conscientiousness moderates the relationship between discrimination and positive affect among aging workers. Everyday discrimination was directly positively correlated with presenteeism. In addition, discrimination increased presenteeism by reinforcing negative affect among aging workers and reduced presenteeism by increasing positive affect. In addition, aging workers with low conscientiousness were more likely than those with high conscientiousness to suppress positive affects when faced with daily discrimination.

Our empirical research showed that perceived everyday discrimination suppressed positive affect and reinforced negative affect among aging workers. Previous research focused mostly on affect in work-related scenarios. Some studies found that criticism, work pressure, and other stressful events had a significant negative correlation with positive affect and a significant positive correlation with negative affect [98,99]. The present study explored the impact of perceived everyday discrimination—a frequent, negative experience of stress that is difficult to address—on the affects of aging workers. Ultimately, our results were similar to those of previous studies of the influence of stressful events on affect. To our knowledge, because stressful events often have varying impacts on different affects, many researchers analyzed positive and negative affects, as this yields more abundant paths to affects. We found that perceived everyday discrimination among aging workers suppresses positive affect and increases negative affect, which is of great practical significance. Policymakers should be made aware of the adverse impact of discrimination on affect. Policies that require fair treatment of aging workers in enterprises should be encouraged, to decrease negative affects in this population.

Another key contribution of this paper is the discovery of a positive relationship between discrimination and presenteeism, a potential counterproductive behavior. Workplace discrimination is strongly discouraged by managers because of its relation to negative work attitudes. Specifically, perceived everyday discrimination is associated with lower job satisfaction [100], lower work engagement [101], and lower organizational commitment [98], which directly lead to decreased working ability among older employees. However, few studies have assessed the indirect and potential effects of discrimination on work ability [97]. Our study has practical significance for increasing the work performance of aging workers. The Australian Productivity Council states that “higher overall economic productivity and labor force participation are key to future economic growth” [49] (2005, p. 303), which indicates that discrimination against aging workers has marked adverse effect on potential productivity loss and could hamper national economic growth. As a society develops, an increasing number of older adults continue working, and many need to be employed after retirement [102]. Thus, managers of enterprises should promote awareness of the effects of discrimination on potential productivity among aging workers by developing employee surveys as well as turnover intention surveys to reduce the risk of discrimination [103]. To decrease presenteeism, policies should be developed that encourage equitable treatment of older workers, combat negative stereotypes of aging workers, reward aging worker role models, increase opportunities for development and training of aging workers, and generate opportunities for positive intergenerational contact [103,104]. Moreover, learning from the traditional Chinese virtue of respecting and caring for the old, society should cultivate a culture of valuing older adults and avoiding perceived everyday discrimination against them. In the same way, because the influence of attendance culture has been regarded as part of the organizations’ context and individuals’ attendance behavior is reciprocally related to the attendance culture, enterprises should also cultivate a culture of valuing aging workers and avoid presenteeism culture. Such an effort, if successful, would help reduce presenteeism among aging employees in enterprises [105,106]. In addition, enhancing the physical, psychological, and interpersonal skills of older workers would help avoid potential productivity loss among them. 

We examined how conscientiousness moderates the relationship between perceived everyday discrimination and positive affect among aging workers. Employees with high and low conscientiousness reported major individual differences in goal achievement, orderliness, and impulse control [78,81,107]. Individuals with high conscientiousness have a greater sense of responsibility and are more proficient at goal-oriented tasks, which results in strong motivation to achieve goals and obey rules [83,108,109]. In contrast, less conscientious workers tend to be less responsible and less interested in goal achievement, which decreases their goal-achievement motivations and intentions to follow rules [108]. Using Emotional Social Information Theory (EASI), researchers explored the effect of 86 leaders’ negative emotions on the performance of 191 employees. Conscientiousness positively moderated the relationship between the leaders’ negative affect and the subordinates’ work performance [74], which is consistent with our findings. Our results indicate that older workers with poor conscientiousness are quick to view discrimination as negative job-related feedback and prejudice from others, which is more likely to result in disappointing work outcomes. Highly conscientious workers are better at self-control and emotional regulation [49,78,83], whereas workers with low conscientiousness are more likely to be affected by fleeting emotions [110]. One study found that individuals with low conscientiousness experience more negative emotions after dispiriting events than do highly conscientious workers [111]. Another study reported that more-conscientious workers recover more easily from negative affect than do less-conscientious workers [78]. Our findings indicate that older workers with low conscientiousness are more likely to suppress positive affects when they experience daily discrimination, which may further reduce their motivation and hamper performance. These results suggest that, to reduce the harmful impact discrimination has on the affects of older workers, managers should attempt to train highly responsible aging workers, award those who are hardworking and goal-oriented, and combine personal goals and organizational goals, particularly through vacations [112,113], as a previous study reported that introduction of vacation leave in workplaces solved work-related problems.

This study has limitations that warrant mention. First, perceived everyday discrimination is a long-term process that may have different effects on the affect and performance of older workers. Causal relationships can only be identified in longitudinal studies. Our study used cross-sectional data to investigate the indirect effect of discrimination on presenteeism in relation to positive and negative affects among older workers. The present data might be useful in future research on the influence of discrimination on presenteeism and in the development of measures to reduce potential loss of productivity among older workers. Secondly, the independent variable of this study was self-reported perceived everyday discrimination among aging workers. Because this type of measurement might not yield adequate ranges for discriminatory incidents as research samples [10], the prevalence and personal consequences of discrimination may be underestimated [10]. Discrimination events are ambiguous and subtle—that is, people may fail to confirm whether the discrimination they encountered every day was the result of their age or gender or some other reason [10]. In addition, some routine incidents may still have adverse effects even if people do not expressly report that they have been ‘discriminated against’ [10,11]. Thus, some subjective cognitive deviation between the questionnaire results and objective conditions is possible. However, because discrimination against older workers leads to considerable adverse effects on work performance, it is particularly important to pay attention to such perceptions of discrimination. Finally, the present sample is from a database of aging workers in the United States. However, in the context of worldwide aging of workers, policymakers must be vigilant regarding the negative impact of discrimination on presenteeism and should focus their efforts on highly conscientious older workers.

## 6. Conclusions

In conclusion, our study indicates that perceived everyday discrimination indirectly affects presenteeism by influencing positive and negative affects among aging workers. Everyday discrimination suppresses positive affects and stimulates negative emotions, which diminishes the mental resources and adversely influences the work performance of older workers, thereby leading to potential loss of productivity. Company managers must therefore demonstrate that they are attuned to emotional changes in older workers by, for example, providing health care and guidance to aging workers experiencing negative affects and by encouraging those with positive affects to increase work output when faced with discrimination.

## Figures and Tables

**Table 1:** Demographic characteristics of sample population (*N* = 2152).

Characteristic	*N*	%
Age (years)		
<50	146	6.8
51–55	533	24.8
56–60	599	27.8
61–65	457	21.2
66–70	212	9.9
>70	204	9.5
**Sex**		
Male	937	43.6
Female	1215	56.4

**Table 2:** Means (SD) for discrimination (D), positive affect (PA), negative affect (NA), presenteeism (P), and conscientiousness (C).

Variable	Item	Mean	SD
Discrimination (1–5)	D1. You are treated with less courtesy or respect than other people.	4.81	1.28
	D2. You receive poorer service than other people at restaurants or stores.	5.33	0.95
	D3. People act as if they think you are not smart.	5.10	1.20
	D4. People act as if they are afraid of you.	5.47	0.97
	D5. You are threatened or harassed.	5.69	0.70
	D6. You receive poorer service or treatment than other people from doctors or hospitals.	5.75	0.62
Positive Affect (1–12)	PA1. Enthusiastic	2.38	1.10
	PA2. Active	2.30	1.10
	PA3. Proud	2.32	1.16
	PA4. Interested	2.13	1.04
	PA5. Happy	1.99	0.94
	PA6. Attentive	2.27	1.05
	PA7. Content	2.29	1.08
	PA8. Inspired	2.59	1.10
	PA9. Hopeful	2.25	1.04
	PA10. Alert	2.03	0.95
	PA11. Calm	2.29	0.98
	PA12. Excited	2.61	1.09
Negative Affect (1–12)	NA1. Afraid	4.45	0.81
	NA2. Upset	3.80	0.96
	NA3. Guilty	4.46	0.82
	NA4. Scared	4.41	0.84
	NA5. Frustrated	3.56	1.11
	NA6. Bored	4.10	0.98
	NA7. Hostile	4.54	0.78
	NA8. Jittery	4.51	0.81
	NA9. Ashamed	4.68	0.71
	NA10. Nervous	4.14	0.95
	NA11. Sad	4.04	0.96
	NA12. Distressed	4.16	0.97
Conscientiousness (1–10)	C1. Reckless	3.52	0.70
	C2. Organized	3.16	0.82
	C3. Responsible	3.78	0.49
	C4. Hardworking	3.78	0.75
	C5. Self-disciplined	3.32	0.74
	C6. Careless	3.40	0.75
	C7. Impulsive	2.77	0.86
	C8. Cautious	3.15	0.76
	C9. Thorough	3.22	0.77
	C10. Thrifty	2.94	0.87
Presenteeism (1–4)	P1. How many points would you give your current ability to work?	8.57	1.63
	P2. Thinking about the physical demands of your job, how do you rate your current ability to meet those demands?	8.49	1.80
	P3. Thinking about the mental demands of your job, how do you rate your current ability to meet those demands?	8.70	1.57
	P4. Thinking about the interpersonal demands of your job, how do you rate your current ability to meet those demands?	8.56	1.63

**Table 3:** Measures of local fit for the modified model.

	Cronbach α	Composite Reliability	Average Variance Extracted
Threshold for Acceptable Fit	≥0.60	≥0.60	≥0.50
Positive Affect	0.93	0.93	0.53
Negative Affect	0.90	0.91	0.47
Discrimination	0.83	0.87	0.52
Presenteeism	0.89	0.92	0.74
Conscientiousness	0.67	0.85	0.39

**Table 4:** Intercorrelations between discrimination (PED), positive affect (PA), negative affect (NA), presenteeism (PAW), and conscientiousness (C).

Variables (Mean, SD)	Item				
	PED	PA	NA	PAW	C
PED (5.36, 0.70)	1	-	-	-	-
PA (2.29, 0.76)	−0.22 ***	1	-	-	-
NA (4.24, 0.61)	0.41 ***	−0.45 ***	1	-	-
PAW (8.58, 1.42)	0.22 ***	−0.34 ***	0.28 ***	1	-
C (3.30, 0.38)	−0.20 ***	0.34 ***	−0.27 ***	−0.27 ***	1

Note: *** *p* < 0.001. Abbreviations: P, presenteeism; D, discrimination; PA, positive affect; NA, negative affect: C, conscientiousness.

**Table 5:** Regression of positive affect on discrimination, conscientiousness, and their interaction.

	Outcome	Variable	β	SE	CI
R^2^ = 0.1450	Positive Affect	Gender	0.0656 *	0.0311	[0.0046, 0.1267]
		Discrimination	0.1812 ***	0.0229	[0.1362, 0.2261]
		Conscientiousness	−0.6426 ***	0.0413	[−0.7236, −0.5616]
∆R^2^ = 0.0018		Positive Affect * Conscientiousness	0.1023 *	0.0485	[0.0072, 0.1973]

Note: SE, standard error; CI, confidence interval; *β*, regression coefficient. *** *p* < 0.001, * 0.01< *p* < 0.05.

**Table 6:** Indirect effects of discrimination on positive affect, by level of conscientiousness.

Variable			BC 1000 BOOT		
Conscientiousness		Positive Affect			
		IND	SE	LLCI (95%)	ULCI (95%)
Low	1.3130	0.1420	0.0264	0.0901	0.1938
Mean	1.6961	0.1812	0.0229	0.1362	0.2261
High	2.0792	0.2204	0.0323	0.1571	0.2836

Note: Coefficients represent specific indirect effects and standard errors at different values of conscientiousness, and the lower and upper bounds of 95% BC bootstrap confidence intervals for that effect, with 1000 bootstrap samples. Low signifies values at 1 SD below the mean, mean signifies values at the mean, and high signifies values at 1 SD above the mean. Abbreviations: IND, indirect effects; LLCI, lower level of confidence interval; ULCI, upper level of confidence interval.

## References

[1] Earl C., Taylor P. (2016). Discriminatory practices of older workers in an ageing residential care workforce. Int. J. Work Innov..

[2] Taylor P., Mcloughlin C., Earl C. (2018). Everyday discrimination in the Australian workplace: Assessing its prevalence and age and gender differences. Australas. J. Ageing.

[3] Lewis T.T., Barnes L.L., Bienias J.L., Lackland D.T., Evans D.A., Mendes de Leon C.F. (2009). Perceived discrimination and blood pressure in older African American and white adults. J. Gerontol. Med. Sci..

[4] Pascoe E.A., Smart R.L. (2009). Perceived discrimination and health: A meta-analytic review. Psychol. Bull..

[5] Williams D.R., Mohammed S.A. (2009). Discrimination and racial disparities in health: Evidence and needed research. J. Behav. Med..

[6] Barnes L.L., De Leon C.F., Wilson R.S., Bienias J.L., Bennett D.A., Evans D.A. (2004). Racial differences in perceived discrimination in a community population of older Blacks and Whites. J. Aging Health.

[7] Hwang W.C., Goto S. (2008). The impact of perceived racial discrimination on the mental health of Asian American and Latino college students. Cult. Divers. Ethn. Minority Psychol..

[8] Sutin A.R., Stephan Y., Carretta H., Terracciano A. (2015). Perceived Discrimination and Physical, Cognitive, and Emotional Health in Older Adulthood. Am. J. Geriatr. Psychiatry.

[9] Kuhn P., Shen K. (2013). Gender Discrimination in Job Ads: Evidence from China. Q. J. Econ..

[10] Deitch E.A., Barsky A., Butz R.M., Chan S., Brief A.P., Bradley J.C. (2003). Subtle yet Significant: The Existence and Impact of Everyday Racial Discrimination in the Workplace. Hum. Relat..

[11] Taylor P., Mcloughlin C., Meyer D., Brooke E. (2013). Everyday discrimination in the workplace, job satisfaction and psychological wellbeing: Age differences and moderating variables. Ageing Soc..

[12] Swim J.K., Hyers L.L., Cohen L.L., Ferguson M.J. (2001). Everyday sexism: Evidence for its incidence, nature, and psychological impact from three daily diary studies. J. Soc. Issues.

[13] O’Loughlin K., Kendig H., Hussain R., Cannon L. (2017). Age discrimination in the workplace: The more things change. Australas. J. Ageing.

[14] Isla R., Dylan K., Cesar D.O., Demakakos P., Steptoe A. (2014). Perceived age discrimination in older adults. Age Ageing.

[15] Sutin A.R., Stephan Y., Grzywacz J.G., Robinson E., Daly M., Terracciano A. (2016). Perceived weight discrimination, changes in health, and daily stressors. Obesity.

[16] Steffens M.C., Niedlich C., Ehrke F., Köllen T. (2016). Discrimination at work on the basis of sexual orientation: Subjective experience, experimental evidence, and interventions. Sexual Orientation and Transgender Issues in Organizations—Global Perspectives on LGBT Workforce Diversity.

[17] Phillips D.R., Siu O., Hedge J.W., Borman W.W.C. (2012). Global Aging and Aging Workers. The Oxford Handbook of Work and Aging.

[18] United Nations Global Issues: Aging. https://www.un.org/en/sections/issues-depth/ageing/index.html.

[19] Han J., Richardson V.E. (2015). The relationships among perceived discrimination, self-perceptions of aging, and depressive symptoms: A longitudinal examination of age discrimination. Aging Ment. Health.

[20] Richard W., Johnson D.N. (1996). Age Discrimination, Job Separation, and Employment Status of Older Workers: Evidence from Self-Reports. J. Hum. Resour..

[21] Fraccaroli F., Deller J. (2015). Work, Aging, and Retirement in Europe: Introduction to the Special Issue. Work Aging Retire..

[22] Zacher H., Griffin B. (2015). Work, Aging, and Retirement in Australia: Introduction to the Special Issue. Work Aging Retire..

[23] Lee Y., Bierman A. (2018). A Longitudinal Assessment of Perceived Discrimination and Maladaptive Expressions of Anger Among Older Adults: Does Subjective Social Power Buffer the Association. J. Gerontol. B Psychol. Sci. Soc. Sci..

[24] Szinovacz M.E. (2011). Introduction: The Aging Workforce: Challenges for Societies, Employers, and Older Workers. J. Aging Soc. Policy.

[25] Weiss H.M., Cropanzano R. (1996). Affective Events Theory: A theoretical discussion of the structure, causes and consequences of affective experiences at work. Res. Organ. Behav..

[26] Lam W., Chen Z. (2012). When I put on my service mask: Determinants and outcomes of emotional labor among hotel service providers according to affective event theory. Int. J. Hosp. Manag..

[27] Comasdíaz L., Greene B. (1994). Women of color: Integrating ethnic and gender identities in psychotherapy. J. Marital Fam. Ther..

[28] Essed P. (1991). Understanding Everyday Racism: An Interdisciplinary Theory. Sage Series on Race and Ethnic Relations. Contemp. Sociol..

[29] Salgado de Snyder V.N. (1986). Mexican immigrant women: The relationship of ethnic loyalty, self-esteem, social support and satisfaction to acculturative stress and depressive symptomatology. Diss. Abstr. Int..

[30] Russell J.A., Feldman B.L. (1999). Core affect, prototypical emotional episodes, and other things called emotion: Dissecting the elephant. J. Personal. Soc. Psychol..

[31] Watson D., Clark L.A., Tellegen A. (1988). Development and validation of brief measures of positive and negative affect: The PANAS scales. J. Personal. Soc. Psychol..

[32] Pressman S.D., Cohen S. (2005). Does positive affect influence health?. Psychol. Bull..

[33] Gross J.J., John O.P. (2003). Individual differences in two emotion regulation processes: Implications for affect, relationships, and well-being. J. Personal. Soc. Psychol..

[34] Jackson K.F., Yoo H.C., Guevarra R., Harrington B.A. (2012). Role of identity integration on the relationship between perceived racial discrimination and psychological adjustment of multiracial people. J. Couns. Psychol..

[35] Morrison T.G., Bishop C.J., Morrison M.A., Parker-Taneo K. (2016). A Psychometric Review of Measures Assessing Discrimination against Sexual Minorities. J. Homosex..

[36] Miraglia M., Johns G. (2015). Going to Work Ill: A Meta-Analysis of the Correlates of Presenteeism and a Dual-Path Model. J. Occup. Health Psychol..

[37] Johns G. (2010). Presenteeism in the workplace: A review and research agenda. J. Organ. Behav..

[38] Ruhle S.A., Breitsohl H., Aboagye E., Baba V., Biron C., Leal C.C., Dietz C., Ferreira A.I., Gerich J., Johns G. (2019). “To work, or not to work, that is the question”—Recent trends and avenues for research on presenteeism. Eur. J. Work Organ. Psychol..

[39] Lohaus D., Habermann W. (2019). Presenteeism: A review and research directions. Hum. Resour. Manag. Rev..

[40] Johansen V. (2018). Motives for sickness presence among students at secondary school: A cross-sectional study in five European countries. BMJ Open..

[41] Karanika-Murray M., Cooper C.L., Cooper C.L., Lu L. (2018). Presenteeism: An introduction to a prevailing global phenomenon. Presenteeism at Work.

[42] Burton W.N., Chen C.Y., Li X., Schultz A.B., Abrahamsson H. (2014). The association of self-reported employee physical activity with metabolic syndrome, health care costs, absenteeism, and presenteeism. J. Occup. Environ. Med..

[43] Zhou Q., Martinez L.F., Ferreira A.I., Rodrigues P. (2016). Supervisor support, role ambiguity and productivity associated with presenteeism: A longitudinal study. J. Bus. Res..

[44] Cooper C.L., Lu L. (2016). Presenteeism as a global phenomenon: Unraveling the psychosocial mechanisms from the perspective of social cognitive theory. Cross Cult. Strateg. Manag..

[45] Yang T., Guo Y., Ma M., Li Y., Tian H., Deng J. (2017). Job Stress and Presenteeism among Chinese Healthcare Workers: The Mediating Effects of Affective Commitment. Int. J. Environ. Res. Public Health.

[46] Yang T., Shen Y.M., Zhu M. (2016). Effects of Co-Worker and Supervisor Support on Job Stress and Presenteeism in an Aging Workforce: A Structural Equation Modelling Approach. Int. J. Environ. Res. Public Health.

[47] Yang T., Ma M., Zhu M. (2018). Challenge or hindrance: Does job stress affect presenteeism among Chinese healthcare workers. J. Occup. Health.

[48] Yang T., Lei R., Jin X., Li Y., Sun Y., Deng J. (2019). Supervisor Support, Coworker Support and Presenteeism among Healthcare Workers in China: The Mediating Role of Distributive Justice. Int. J. Environ. Res. Public Health.

[49] Cho Y.S., Park J.B., Lee K.J., Min K.B., Baek C.I. (2016). The association between Korean workers’ presenteeism and psychosocial factors within workplaces. Ann. Occup. Environ. Med..

[50] Min J.Y., Park S.G., Kim S.S., Min K.B. (2014). Workplace injustice and self-reported disease and absenteeism in South Korea. Am. J. Ind. Med..

[51] Tsai H.C., Thompson E.A. (2015). Effects of Social Determinants on Chinese Immigrant Food Service Workers? Work Performance and Injuries. J. Occup. Environ. Med..

[52] Bowling N.A., Beehr T.A. (2006). Workplace harassment from the victim’s perspective: A theoretical model and meta-analysis. J. Appl. Psychol..

[53] Willness C.R., Steel P., Lee K. (2010). A meta-analysis of the antecedents and consequences of workplace sexual harassment. Pers. Psychol..

[54] Barsade S.G., Gibson D.E. (2012). Group Affect: Its Influence on Individual and Group Outcomes. Curr. Dir. Psychol. Sci..

[55] Brief A.P., Weiss H.M. (2002). Organizational behavior: Affect in the workplace. Annu. Rev. Psychol..

[56] Judge T.A., Scott B.A., Ilies R. (2006). Hostility, job attitudes, and workplace deviance: Test of a multilevel model. J. Appl. Psychol..

[57] Paul L., Gerhard M.S., Andrew N. (2010). The impact of pain on labor force participation, absenteeism and presenteeism in the European Union. J. Med. Econ..

[58] Ravyts S.G., Dzierzewski J.M., Raldiris T., Perez E. (2018). Sleep and pain interference in individuals with chronic pain in mid- to late-life: The influence of negative and positive affect. J. Sleep Res..

[59] Alex J.Z., Mary C.D., Bruce W.S. (2005). Emotions, Personality, and Health: Introduction to the Special Issue. J. Personal..

[60] Thundiyil T.G., Chiaburu D.S., Li N., Wagner D.T. (2016). Joint effects of creative self-efficacy, positive and negative affect on creative performance. Chin. Manag. Stud..

[61] Thøgersen-Ntoumani C., Loughren E.A., Kinnafick F.E., Taylor I.M., Duda J.L., Fox K.R. (2015). Changes in work affect in response to lunchtime walking in previously physically inactive employees: A randomized trial. Scand. J. Med. Sci. Sports.

[62] Ambrona T., Belén L.P. (2014). A Longitudinal Analysis of the Relationship between Positive and Negative Affect and Health. Psychology.

[63] Fiori M., Bollmann G.R. (2015). Exploring the path through which career adaptability increases job satisfaction lowers job stress: The role of affect. J. Vocat. Behav..

[64] Diefendorff J.M., Mehta K. (2007). The relations of motivational traits with workplace deviance. J. Appl. Psychol..

[65] Johnson R.E., Tolentino A.L., Rodopman O.B., Cho E. (2010). We (sometimes) know not how we feel: Predicting job performance with an implicit measure of trait affectivity. Pers. Psychol..

[66] Shockley K.M., Ispas D., Rossi M.E., Levine E.L. (2012). A meta-analytic investigation of the relationship between states affect, discrete emotions, and job performance. Hum. Perform..

[67] Aquino K., Lewis M.U., Bradfield M. (1999). Justice Constructs, negative affectivity, and employee deviance: A proposed model and empirical test. J. Organ. Behav..

[68] Ferreira A.I., da Costa Ferreira P., Cooper C.L., Oliveira D. (2019). How daily negative affect and emotional exhaustion correlates with work engagement and presenteeism-constrained productivity. Int. J. Stress Manag..

[69] Isen A.M., Daubman K.A., Nowicki G.P. (1987). Positive affect facilitates creative problem-solving. J. Personal. Soc. Psychol..

[70] Gable S.L., Reis H.T., Elliot A.J. (2003). Evidence for bivariate systems: An empirical test of appetition and aversion across domains. J. Res. Personal..

[71] Skarlicki D.P., Folger R., Tesluk P. (1999). Personality as a moderator in the relationship between fairness and retaliation. Acad. Manag. J..

[72] Barrick M.R., Mount M.K., Judge T.A. (2001). Personality and performance at the beginning of the new millennium: What do we know and where do we go next. Int. J. Sel. Assess..

[73] Chi N.W., Tsai W.C., Tseng S.M. (2013). Customer negative events and employee service sabotage: The mediating role of employee hostility and the moderating roles of personality and group affective tone. Work Stress.

[74] Chi N.W., Ho T.R. (2014). Understanding When Leader Negative Affectal Expression Enhances Follower Performance. Acad. Manag. Annu. Meet. Proc..

[75] Hengartner M.P., Linden D., Bohleber L. (2017). Big Five Personality Traits and the General Factor of Personality as Moderators of Stress and Coping Reactions Following an Emergency Alarm on a Swiss University Campus. Stress Health.

[76] Husnain M., Qureshi I., Fatima T., Akhtar W. (2016). The Impact of Electronic Word-of-Mouth on Online Impulse Buying Behavior: The Moderating role of Big 5 Personality Traits. J. Acc. Mark.

[77] Sutin A.R., Stephan Y., Luchetti M., Artese A., Oshio A., Terracciano A. (2016). The Five-Factor Model of Personality and Physical Inactivity: A Meta-Analysis of 16 Samples. J. Res. Personal..

[78] Javaras K.N., Schaefer S.M., Van Reekum C.M. (2012). Conscientiousness predicts greater recovery from negative emotion. Emotion.

[79] Lin W., Ma J., Wang L., Wang M.O. (2015). A double-edged sword: The moderating role of conscientiousness in the relationships between work stressors, psychological strain, and job performance. J. Organ. Behav..

[80] Costa P.T., McCrae R.R. (1992). Revised NEO Personality Inventory and New Five-Factor Inventory: Professional Manual.

[81] Roberts B.W., Chernyshenko O.S., Stark S., Goldberg L.R. (2005). The structure of conscientiousness: An empirical investigation based on seven major personality questionnaires. Pers. Psychol..

[82] Fayard J.V., Roberts B.W., Robbins R.W., Watson D. (2012). Uncovering the affective core of conscientiousness: The role of self-conscious emotions. J. Personal..

[83] George J.M., Zhou J. (2001). When openness to experience and conscientiousness are related to creative behavior: An interactional approach. J. Appl. Psychol..

[84] Shultz K.S., Wang M. (2011). Psychological perspectives on the changing nature of retirement. Am. Psychol..

[85] Soldo B.J., Hurd M.D., Rodgers W.L. (1997). Asset and Health Dynamics among the Oldest Old: An Overview of the AHEAD Study. J. Gerontol. B Psychol. Sci. Soc. Sci..

[86] Williams D.R., Yu Y., Jackson J.S., Anderson N.B. (1997). Racial differences in physical and mental health: Socio-economic status, stress and discrimination. J. Health Psychol..

[87] Ilmarinen J., Rantanen J. (1999). Promotion of Work Ability during Ageing. Am. J. Ind. Med..

[88] Vänni K., Virtanen P., Luukkaala T. (2012). Relationship between perceived work ability and productivity loss. Int. J. Occup. Saf. Erg..

[89] Smith J., Fisher G., Ryan L., Clarke P., House J., Weir D. (2017). Psychosocial and Lifestyle Questionnaire 2006–2016 Documentation Report Core Section LB.

[90] Carstensen L.L., Pasupathi M., Mayr U., Nesselroade J.R. (2000). Affectal experience in everyday life across the adult life span. J. Personal. Soc. Psychol..

[91] Ong A.D., Edwards L.M., Bergeman C.S. (2006). Hope as a source of resilience in later adulthood. Personal. Individ. Differ..

[92] Lachman M.E., Weaver S.L. (1997). The Midlife Development Inventory (MIDI) Personality Scales: Scale Construction and Scoring.

[93] Preacher K.J., Hayes A.F. (2004). SPSS and SAS procedures for estimating indirect effects in simple mediation models. Behav. Res. Methods Instrum. Comput..

[94] Williams J., Mackinnon D.P. (2008). Resampling and distribution of the product methods for testing indirect effects in complex models. Struct. Equ. Model.

[95] Jing Q., Bin W., Zhuo H., Song B. (2017). Ethical Leadership, Leader-Member Exchange and Feedback Seeking: A Double-Moderated Mediation Model of Emotional Intelligence and Work-Unit Structure. Front. Psychol..

[96] Hayes A.F., Preacher K.J. (2014). Statistical mediation analysis with a multicategorical independent variable. Br. J. Math. Stat. Psychol..

[97] Hayes A.F. (2013). Introduction to Mediation, Moderation, and Conditional Process Analysis: A Regression-Based Approach.

[98] Dunkley D.M., Ma D., Lee I.A., Preacher K.J., Zuroff D.C. (2014). Advancing complex explanatory conceptualizations of daily negative and positive affect: Trigger and maintenance coping action patterns. J. Couns. Psychol..

[99] Dunkley D.M., Lewkowski M., Lee I.A., Preacher K.J., Zuroff D.C., Berg J.L., Foley J.E., Myhr G., Westreich R. (2017). Daily Stress, Coping, and Negative and Positive Affect in Depression: Complex Trigger and Maintenance Patterns. Behav. Ther..

[100] Redman T., Snape E. (2006). The consequences of perceived age discrimination amongst older police officers: Is social support a buffer. Br. J. Manag..

[101] Rabl T., María Del Carmen T. (2013). How German employees of different ages conserve resources: Perceived age discrimination and affective organizational commitment. Int. J. Hum. Resour. Manag..

[102] Australian Productivity Commission (2005). Economic Implications of an Ageing Australia. Productivity Commission, Government of Australia Research Reports. http://www.pc.gov.au/data/assets/pdf.file/0020/69401/ageing.pdf.

[103] Goldman B.M., Gutek B.A., Stein J.H., Lewis K. (2006). Employment discrimination in organizations: Antecedents and consequences. J. Manag..

[104] Kunze F., Boehm S.A., Bruch H. (2011). Age diversity, age discrimination climate and performance consequences—A cross organizational study. J. Organ. Behav..

[105] Dew K., Keefe V., Small K. (2005). Choosing to work when sick: Workplace presenteeism. Soc. Sci. Med..

[106] Ruhle S.A., Süß S. (2019). Presenteeism and Absenteeism at Work—An Analysis of Archetypes of Sickness Attendance Cultures. J. Bus. Psychol..

[107] Henry H., Zacher H., Desmette D. (2015). Reducing age bias and turnover intentions by enhancing intergenerational contact quality in the workplace: The role of opportunities for generativity and development. Work Aging Retire..

[108] Rothbart M., Sheese B., Gross J. (2007). Temperament and affect regulation. Handbook of Affect Regulation.

[109] Barrick M.R., Mount M.K., Strauss J.P. (1993). Conscientiousness and performance of sales representatives: Test of the mediating effects of goal setting. J. Appl. Psychol..

[110] Hollenbeck J.R., Klein H.J. (1987). Goal Commitment and the Goal-Setting Process: Problems, Prospects, and Proposals for Future Research. J. Appl. Psychol..

[111] Ilies R., Scott B.A., Judge T.A. (2006). The interactive effects of personal traits and experienced states on intraindividual patterns of citizenship behavior. Acad. Manag. J..

[112] Fakih A. (2014). Vacation Leave, Work Hours, and Wages: New Evidence from Linked Employer-Employee Data. LABOUR.

[113] Fakih A. (2018). What determines vacation leave? The role of gender. Bull. Econ. Res..

